# Expression of E-cadherin and specific CXCR3 isoforms impact each other in prostate cancer

**DOI:** 10.1186/s12964-019-0489-1

**Published:** 2019-12-12

**Authors:** Bo Ma, Ahmad Khazali, Hanshuang Shao, Yuhan Jiang, Alan Wells

**Affiliations:** 1 Department of Pathology, University of Pittsburgh, S713 Scaife Hall, 3550 Terrace St, Pittsburgh, PA 15261 USA; 2 Jiangsu Center for the Collaboration and Innovation of Cancer Biotherapy, Cancer Institute, Xuzhou Medical University, 84 Huaihai Xi Road, Quanshan, Xuzhou, Jiangsu 221002 People’s Republic of China; 3 Pittsburgh VA Medical Center, VA Pittsburgh Healthcare System, Pittsburgh, USA; 4 Department of Molecular Medicine, University of Malaya, Kuala Lumpur, Malaysia; 5 School of Medicine, Tsinghua University, Beijing, China; 6 McGowan Institute for Regenerative Medicine, University of Pittsburgh, Pittsburgh, PA USA; 7 UPMC Hillman Cancer Center, University of Pittsburgh, Pittsburgh, PA USA

**Keywords:** Prostate cancer; metastasis, CXCR3 variant, CXCR3-B, E-cadherin, EMT, MErT

## Abstract

**Background:**

Carcinoma cells shift between epithelial and mesenchymal phenotypes during cancer progression, as defined by surface presentation of the cell-cell cohesion molecule E-cadherin, affecting dissemination, progression and therapy responsiveness. Concomitant with the loss of E-cadherin during the mesenchymal transition, the predominant receptor isoform for ELR-negative CXC ligands shifts from CXCR3-B to CXCR3-A which turns this classical G-protein coupled receptor from an inhibitor to an activator of cell migration, thus promoting tumor cell invasiveness. We proposed that CXCR3 was not just a coordinately changed receptor but actually a regulator of the cell phenotype.

**Methods:**

Immunoblotting, immunofluorescence, quantitative real-time PCR and flow cytometry assays investigated the expression of E-cadherin and CXCR3 isoforms. Intrasplenic inoculation of human prostate cancer (PCa) cells with spontaneous metastasis to the liver analyzed E-cadherin and CXCR3-B expression during cancer progression in vivo.

**Results:**

We found reciprocal regulation of E-cadherin and CXCR3 isoforms. E-cadherin surface expression promoted CXCR3-B presentation on the cell membrane, and to a lesser extent increased its mRNA and total protein levels. In turn, forced expression of CXCR3-A reduced E-cadherin expression level, whereas CXCR3-B increased E-cadherin in PCa. Meanwhile, a positive correlation of E-cadherin and CXCR3-B expression was found both in experimental PCa liver micro-metastases and patients’ tissue.

**Conclusions:**

CXCR3-B and E-cadherin positively correlated in vitro and in vivo in PCa cells and liver metastases, whereas CXCR3-A negatively regulated E-cadherin expression. These results suggest that CXCR3 isoforms may play important roles in cancer progression and dissemination via diametrically regulating tumor’s phenotype.

## Background

Metastasis is the main cause of cancer related death [1]. However, it is an inefficient process as the tumor cells need to accomplish several sequential steps to successfully disseminate [2]. In the early steps of metastasis, cancer cells undergo a cancer-associated epithelial-to-mesenchymal transition (cEMT) that disrupts cell-cell contacts, increases cell motility and allows the cancer cells to invade through the surrounding stroma and intravasate into the blood circulation. Upon extravasation, metastatic cancer cells revert via a second step of a cancer-associated mesenchymal-to-epithelial reverting transition (cMErT) to form heterotypic E-cadherin connection with the parenchyma of the secondary organs that transmit survival signals to allow cancer cells seeding to survive the hostile ectopic environment [3–5].

Central to both EMT and MErT is the tumor suppressor cell-cell cohesion transmembrane protein E-cadherin [6]. Down-regulation of E-cadherin from the membrane releases tumor cells from primary sites allowing metastasis. However, cancer cells phenotypic plasticity is transient and reversible [7]. Re-expression of E-cadherin during cMErT, driven in part by the organ’s parenchyma [8, 9], is essential for efficient metastasis. Furthermore, signaling through the liganded E-cadherin via the cytosolic Akt, Erk and Jak pathways also provides the disseminated cells with a survival advantage in the face of local cell death factors or chemotherapy [4, 10, 11] . Moreover, micro-metastases express E-cadherin, whereas a secondary cEMT, partially driven by local inflammation factors, is required for tumors outgrowth and emergence [12–14].

CXCR3 is a G-protein coupled receptor which binds four ELR-negative CXC chemokines, including CXCL9 (MIG), CXCL10 (IP-10), CXCL11 (IP-9/I-TAC) and CXCL4L1 [15, 16]. Activation of this receptor is crucial for chemotaxis induction during immune responses, vascular development and wound repair [17–19]. In the context of cancer, CXCR3 was found upregulated in many primary and metastatic tumors such as breast, prostate, colon, colorectal, melanoma and ovarian cancer, and associated with poor prognosis [20–24]. In the tumor organ, CXCR3 and its ligands are expressed on the tumor cells, stromal cells, vessels and recruited leukocytes, with most all of these cells also producing various ligands [16]. Consequently, CXCR3 directly or indirectly involves in tumor progression by regulating tumor outgrowth, migration, invasion, angiogenesis and immunity. CXCR3-A and CXCR3-B, the two isoforms of CXCR3 induce opposite physiological and pathological functions. Generally, CXCR3-A, the predominant form in hematopoietic cells, appears to mediate “go” signaling via promoting cell proliferation, survival, chemotaxis, invasion and metastasis, while CXCR3-B, the main form on formed elements including epithelial cells, appears to mediate “stop” signaling via promoting growth suppression, apoptosis and vascular involution [16]. Thus, aberrant expression of CXCR3-A or CXCR3-B could affect tumor progression.

We previously investigated the roles of CXCR3 isoforms in prostate cancer (PCa) and found that the predominant CXCR3 isoform shifts from CXCR3-B to CXCR3-A during malignancy [25]. Meanwhile, E-cadherin is dynamically regulated during tumor progression. Although the expression of both CXCR3 isoforms and E-cadherin are altered along with the phenotypic plasticity, whether these are merely coordinated changes or functionally linked to each other has not been investigated. Interestingly, reduction of CXCR3-B expression has been found in two classic PCa cell lines DU145 and PC3 cells, which show reduced surface E-cadherin compared to a less mesenchymal PCa line, LNCaP, and immortalized normal prostate cell line RWPE1 [25]. We hypothesized that CXCR3 isoforms differently affect E-cadherin expression.

We utilized prostate cancer DU145 and PC3 cells, reported to express varying level of E-cadherin, to test our hypothesis. First, we found that EGFR inhibitor induced epithelial transitioned DU145 cells, as well as the DU145 cells harboring high E-cadherin sub-line (DU-H), presented more CXCR3-B on the cell membrane and elevated its downstream target cAMP when compared to DU145 E-cadherin low (DU-L) cells. Additionally, forced expression of CXCR3A in DU-H cells reduced E-cadherin protein level whereas overexpression of CXCR3-B in DU-L cells increased E-cadherin surface expression. Moreover, exposure to CXCR3 ligand IP-10 or its inhibitor AMG-487 differentially modulated E-cadherin expression in DU-H cells. We also observed significant correlation between CXCR3-B and E-cadherin in liver metastases in an experimental mouse model of spontaneous PCa metastasis. These results suggest that as part of driving or inhibiting cell migration, CXCR3 isoform selection and E-cadherin presentation form a regulatory loop, and synergistically impact cellular phenotype.

## Methods

### Cell culture

Parental DU145 E-cadherin^low^ (DU-L) and E-cadherin^high^ (DU-H) cells [11] were cultured in DMEM with 4.5 g/L glucose, L-glutamine and sodium pyruvate (Corning) supplemented with 10% FBS and 1% penicillin/streptomycin (Gibco). PC3 cells were cultured in F12K medium (Gibco) supplemented with 10% FBS and 1% penicillin/streptomycin. MDA-MB231 cells were cultured in RPMI-1640 (Gibco) supplemented with 10% FBS and 1% penicillin/streptomycin.

### Transfection

pTarget-CXCR3A and pTarget-CXCR3B were gifts from Dr. P. Romagnani (Florence, Italy) [26]. pCMV-CXCR3B-ddk plasmids were purchased from Origene. Prior to transfection, cells were seeded and cultured for an overnight. Transfection was performed using Lipofectamine-2000 (Invitrogen) according to manufacturer’s protocol for 4–6 h and allowed to recover in complete medium for 24–48 h. CXCR3-B overexpressing cells were subsequently cultured in complete DMEM containing 1 mg/ml G418.

### Immunoblotting

Cancer cells were seeded and cultured until the cells reached 90% confluency. Ice-cold RIPA buffer supplemented with protease and phosphatase inhibitors was used to harvest cell lysates. BCA assay (Thermo Fisher) was performed to determine protein concentration. Proteins were separated using SDS-PAGE and transferred to PVDF membrane (Millipore). Primary antibody incubation was performed at 4 °C for an overnight and secondary antibody was performed at room temperature for an hour. Protein bands were detected using chemiluminescent substrates (Thermo Fisher) on autoradiography films with SRX-101A developer (Konica Minolta).

### Quantitative reverse-transcription PCR (qRT-PCR)

Cells were seeded and cultured until 90% confluency. RNA was harvested using TRIzol reagent (Invitrogen) according to manufacturer’s protocol. Reverse transcription was performed using Quantitect Reverse Transcription kit (Qiagen) and qRT-PCR was performed using Sybr Green PCR Master Mix (Invitrogen) on MX3000P Stratagene machine (Agilent Genomics). The primers used were as follows: CXCR3(forward, fw): 5′-CATCAACTTCTACGCAGGAG-3′; CXCR3(reverse, rv): 5′-AGTCACTGCTGAGCTGGAGG-3′; CXCR3-A fw: 5′- CCCAGCCATGGTCCTTGAGGTGAGTG-3′; CXCR3-A rv: 5′-AAAGAGGAGGCTGTAGAGGGCTGGCAG-3′; CXCR3-B fv: 5′-GGATCACGCAGAAGAATGCGAG-3′; CXCR3-B rv: 5′-GAAGGGGCTGTGTAAAGGCCTG-3′; GAPDH fw: 5′-GAGTCAACGGATTTGGTCGT-3′ GAPDH rv: 5′-TTGATTTTGGAGGGATCTCG-3′; E-cadherin fw: 5′- CTGTGCCCAGCCTCCATGTTTT-3′ and E-cadherin rv: 5′- CTGGATAGCTGCCCATTGCAAGTTA-3′ (E-cadherin reverse). Gene expression fold-change was normalized against GAPDH and calculated using ΔΔCt method.

### Flow cytometry

Cells were cultured overnight in complete DMEM. The following day, cells were detached with enzyme free cell dissociation buffer, washed and fixed with 4% paraformaldehyde (Electron Microscopy Sciences). Following permeabilization with 0.1% Triton X-100 (omitted for cell surface staining) and blocking in 5% BSA buffer, cells were then incubated with primary antibody for 30 min on ice, followed by 30 min secondary antibody incubation with PBS washing between the incubation periods. Sample stained with secondary antibody alone was used a negative control. Samples were run on FACS Calibur (BD Biosciences). Analyses was performed using FlowJo software.

### Immunofluorescence

Cells were cultured on heat-sterilized coverslips. The cells were fixed with 4% paraformaldehyde (Electron Microscopy Sciences), permeabilized with 0.1% Triton-X 100 (Sigma) and blocked with 5% bovine serum albumin (Sigma) for 1 h at room temperature. Cells were then incubated with primary antibodies for an overnight at 4 °C. Then, the cells were rinsed with PBS and incubated with secondary antibodies for an hour at room temperature. Cells were imaged using Olympus Fluoview 1000 microscope.

### Immunohistology

Prior to immunostaining, paraffin-embedded sections were subjected to deparaffinization and antigen retrieval with boiling for 10 min in 0.1 M citrate buffer (with 1 mM EDTA), pH 6.0. The images were acquired using an Olympus Provis microscope. The semi-quantification of DAB OD value was performed using Fiji-ImageJ software. Briefly, liver metastatic tumor nodules were outlined, following with semi-quantification of the staining of CXCR3-B (DAB OD value and the tumor area). The correlation of CXCR3-B and the size of tumor nodules were shown by nonlinear fit line with R-squared value.

### cAMP ELISA

Cultured cells were serum-starved for 24 h. The media were then changed to serum-free DMEM + 100 μM 3-isobutyl-1-methylxanthine (IBMX) with or without 5 μM forskolin for 2 h. Forskolin treatment served as the positive controls. Samples were harvested by incubating the cells with 0.1 M HCl for 10 min at room temperature. Cells were scraped, pipetted several times and pelleted. The supernatants were immediately transferred to − 80 °C. ELISA was performed on 1:2 diluted supernatants according to manufacturer’s protocol (Cayman Chemicals). Raw data were corrected to the blank and non-specific binding (NSB) wells. The data were then plotted with 4-parameter sigmoidal curve using GraphPad Prism (La Jolla, CA).

### Intrasplenic inoculation of human PCa cells in mouse

The animal studies and procedures were approved by the Association for Assessment and Accreditation of Laboratory Animal Care-accredited Institutional Animal Care and Use Committees of Veteran’s Administration Pittsburgh Health System. Seven-week-old male NOD/SCID gamma mice (Jackson Laboratory) were anesthetized with ketamine/xylazine. Long-acting buprenorphine was used as pain reliever. Sterile surgery was performed to expose the spleen. Half a million of cancer cells were injected into the spleen using 27-gauge needle. Running stitch with absorbable suture was performed to close the omentum and metal wound clips were used to close the skin wound. Mice were sacrificed after 5 weeks using a carbon dioxide chamber according to AVMA Guidelines on Euthanasia.

### Matched human prostate tumor and metastases samples

Paired autopsy samples were obtained from the UPMC Hillman Cancer Center and Tissue and Research Pathology/Pitt Biospecimen Core, which is supported in part by award P30CA047904. These included the primary prostate tumors, along with metastases to lung (2 cases), liver (1 case), bone (2 cases) and bone marrow (1 case). The use of these cells and slides was approved by the University of Pittsburgh IRB as exemption 4e.

### Reagents

The following antibodies and reagents were used throughout the study: IP-10 (Peprotech 300–12), AMG487 (Tocris 448,710), mouse anti-human CXCR3-B specific antibody (Proteintech 60,065–1-Ig), mouse-anti human CXCR3 (R&D MAB160, recognizes both CXCR3-A and CXCR3-B), rabbit anti-human GAPDH (Cell Signaling 14C10), rabbit anti-human actin (Sigma A2668), mouse anti-human β-tubulin (Santa Cruz Biotech SC-101527), mouse-anti ddk tag (Origene TA50011–100), mouse anti-human E-cadherin (Invitrogen 135,700)-for immunofluorescence and rabbit anti-human E-cadherin (Cell Signaling 3195)-for immunoblotting and immunohistology.

### Statistical analyses

Statistical analyses for all experiments were performed with using Prism7/8 software (GraphPad, La Jolla, CA). An independent Student’s t-test was used to determine statistical differences between experimental and control values. Fisher’s exact test was used to determine statistical significance of correlation between E-cadherin and CXCR3-B, *P* values <0.05 were considered statistically significant.

## Results

### CXCR3-B cell surface levels correlate with E-cadherin in DU145

**Fig. 1:**
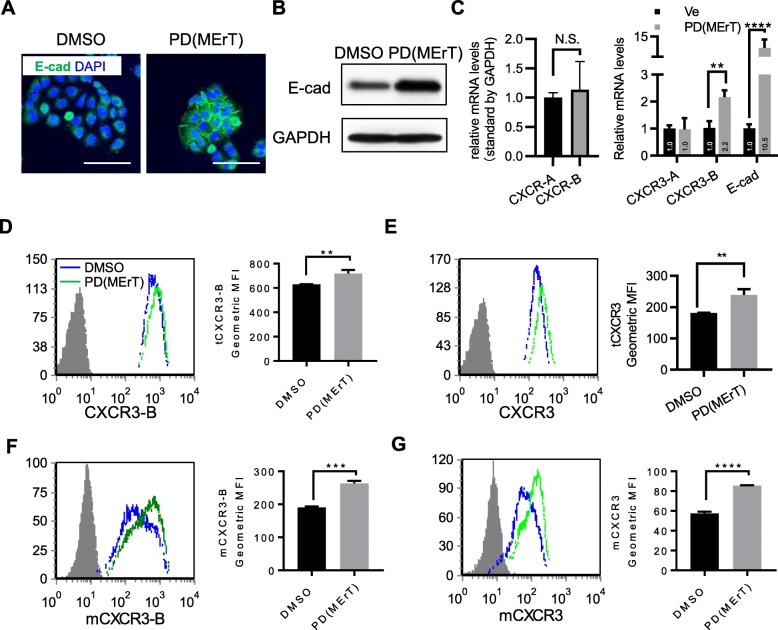
Membrane-presented CXCR3-B is increased in epithelial PCa cells. In (**a-g**), DU145 cells treated with 500 nM PD153035 for 48 h to induce epithelial conversion (PD(MErT)), DMSO was added as control. **a** Immunofluorescence staining of E-cadherin (green) and DAPI (blue). Bar = 25μm. **b** Immunoblot of E-cadherin expression, GAPDH as loading control. **c**) Quantitative real-time PCR analysis. Relative mRNA levels of CXCR3-A, CXCR3-B in DU145 cells (left panel); and CXCR3-A, CXCR3-B and E-cadherin in epithelial converted cells (fight panel); normalized to GAPDH. In (**d-g**), flow cytometry assessments of whole cell level of CXCR3-B (**d**), whole cell level of total-CXCR3 (E), externally-accessible CXCR3-B (F), externally-accessible total-CXCR3 (**g**). The Geometric Mean Fluorescence Intensity(MFI) is on the right panel. Student *t-*test, **, *p* < 0.01; ***, *p* < 0.001; ****, *p* < 0.0001. One representative experiment of at least 3 independent repeats is presented in all panels

To determine whether CXCR3 isoform levels correlate with cancer cell phenotype, we compared the expression of CXCR3 isoforms in PD153035-induced epithelial transitioned DU145 cells [27, 28]. Exposure DU145 (DU145 E-cadherin^low^, DU-L) to 500 nM EGFR inhibitor PD153035 induced E-cadherin expression as previously reported (Fig. 1a and b) [28]. DU145 cells had similar CXCR3-A and CXCR3-B mRNA levels (Fig. 1c, left). No substantial differences of CXCR3-A were noted at the mRNA level between parental and PD-converted cells, while CXCR3-B mRNA levels were increased in epithelial transitioned cells (Fig. 1c, right). Consistently, CXCR3-B and CXCR3 whole cell protein levels were increased to a greater extent in E-cadherin-presenting DU145 cells as determined by flow cytometry (Fig. 1d and e); CXCR3-A protein levels were not determined due to lack of an antibody specific for this isoform by flow. However, flow cytometry analysis on non-permeabilized cells showed significant higher CXCR3-B and total CXCR3 on the membrane for the epithelial-transitioned cells when compared to the parental DU-L (Fig. 1f and g). An immunoblot of CXCR3 is able to distinguish the two isoforms in during synthesis due to the different molecular weight, CXCR3-B increased and CXCR3-A decreased after PD153035 induced DU-L epithelial conversion (Additional file 1: Figure S1a).

**Fig. 2:**
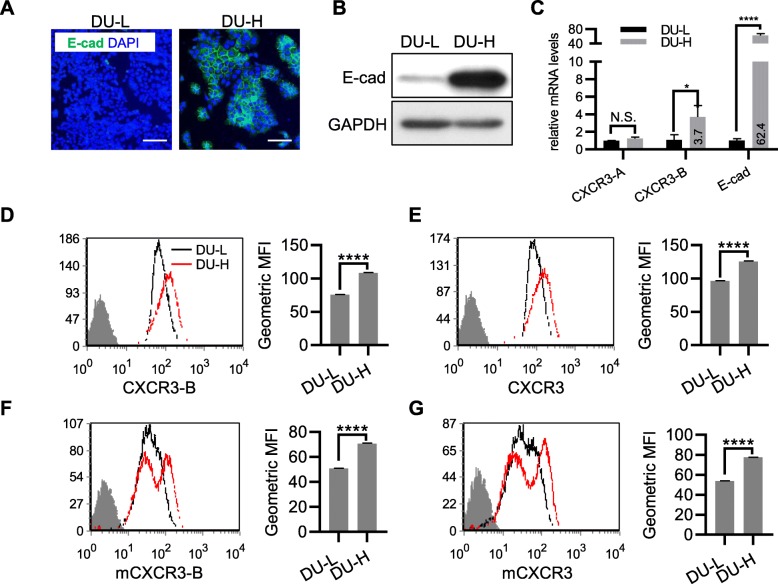
E-cadherin high DU145 sub-line presents higher levels of CXCR3 and CXCR3-B. In (**a**-**g**), DU145 sub-lines with low E-cadherin (DU-L) or high E-cadherin (DU-H). **a** Immunofluorescence staining of E-cadherin (green) and DAPI (blue). Bar = 50μm. **b** Immunoblot of E-cadherin expression, GAPDH as loading control. **c** Quantitative real-time PCR analysis of mRNA levels of CXCR3-A, CXCR3-B and E-cadherin; normalized to GAPDH. In (**d**-**g**), flow cytometry assay of whole cell level of CXCR3-B (**d**), whole cell level of total-CXCR3 (**e**), externally-accessible CXCR3-B (**f**), externally-accessible total-CXCR3 (**g**). The Geometric Mean Fluorescence Intensity (MFI) is on the right panel. Student *t-*test, *, *p* < 0.05; ****, *p* < 0.0001, N.S., non-specific. One representative experiment, of at least 3 independent repeats, is presented in all panels

This was also verified with the sub-lines of DU145 (DU-L and DU145 E-cadherin^high^, DU-H). DU-H in culture established cell-cell contact via E-cadherin heterotypic binding, while DU-L lack membrane E-cadherin and cell-cell contact though still grow in colony (Fig. 2a and b). No obvious differences in CXCR3-A mRNA levels were found between DU-L and DU-H. However, CXCR3-B mRNA was marked higher in DU-H (Fig. 2c). The whole cell protein levels of CXCR3-B and CXCR3 were increased in DU-H (Fig. 2d and e), concomitant with elevated cell surface protein levels (Fig. 2f and g). Immunoblot data demonstrated that CXCR3-B increased in DU-H, while CXCR3-A decreased with comparison to DU-L. Knocking down E-cadherin in DU-H reverse such isoforms switching (Additional file 1: Figure S1b). Additionally, cAMP levels, downstream target of CXCR3-B, were significantly higher in DU-H cells (Additional file 1: Figure S1c), establishing the functionality of the CXCR3-B in these PCa cells.

### Down-regulation of E-cadherin in DU-H decreased CXCR3 and CXCR3-B

**Fig. 3:**
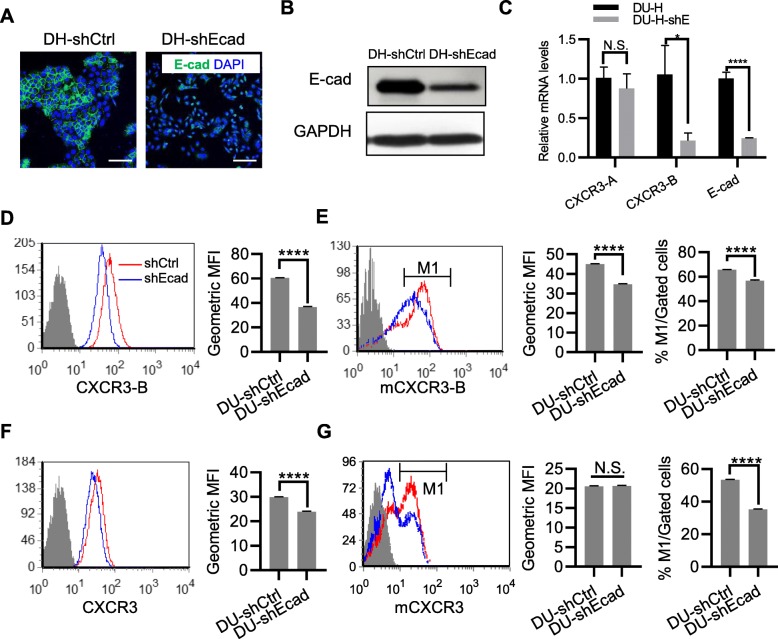
Reduction in E-cadherin decreased CXCR3 expression and membrane presentation. In (**a-g)**, DU145 E-cadherin high sub-line with stable expression of control shRNA (DH-shCtrl) or E-cadherin shRNA (DH-shEcad). **a** Immunofluorescence staining of E-cadherin (green) and DAPI (blue). Bar = 50μm. **b** Immunoblot of E-cadherin expression, GAPDH as loading control. **c** Quantitative real-time PCR analysis of mRNA levels of CXCR3-A, CXCR3-B and E-cadherin; normalized to GAPDH. In (**d-g**), flow cytometry assays of whole cell level of CXCR3-B (**d**), externally-accessible CXCR3-B (**e**), whole cell level of total-CXCR3 (**f**), externally-accessible total-CXCR3 (**g**). The Geometric Mean Fluorescence Intensity (MFI) or percentage of M1 among the gated cells is on the right. Student *t-*test, *, *p* < 0.05; ****, *p* < 0.0001; N.S, non-specific. One representative experiment of at least 3 independent repeats is presented in all panels

To further investigate the regulation of E-cadherin on CXCR3 expression, E-cadherin was stably downregulated by shRNA in DU-H (Fig. 3a and b). This led to the decrease of CXCR3-B mRNA levels (Fig. 3c), but not that of CXCR3-A. Moreover, both whole cell and cell surface CXCR3-B decreased in E-cadherin knocked down DU-H cells, which harbors high intrinsic levels of E-cadherin (Fig. 3d and e). To a lesser extent than CXCR3-B, CXCR3 protein levels were reduced as well (Fig. 3f and g). These findings suggested that E-cadherin regulated the expression and location of CXCR3, and CXCR3-B in particular.

### Forced expression of CXCR3 isoforms differentially regulated E-cadherin

**Fig. 4:**
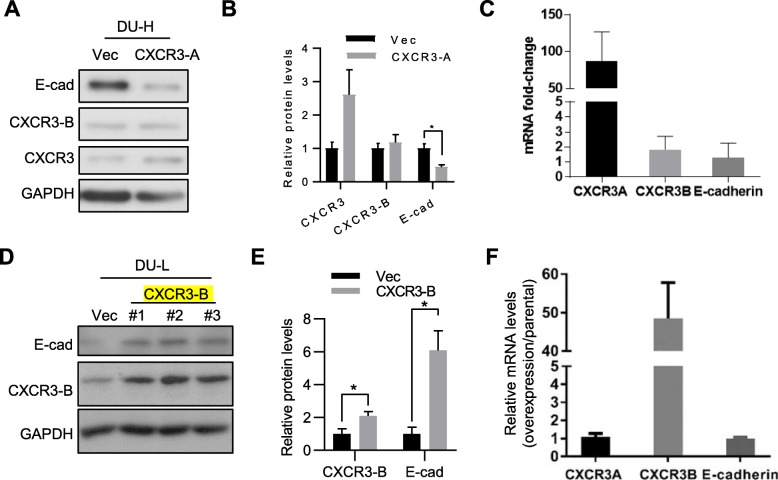
Overexpression of CXCR3 variants diametrically regulate E-cadherin. In (**a**-**c**), transient transfected DU-H cells with empty or CXCR3-A plasmids. **a** Representative immunoblot of E-cadherin, CXCR3-B and CXCR3, GAPDH as loading control. **b** Semi-quantification of protein expression levels by immunoblot band densitometry, student t-test, *, *p* < 0.05, *n* = 4. **c** Quantitative real-time PCR analysis, data shown as ratio of mRNA levels of CXCR3-A/empty plasmids transfections. One representative experiment is presented with 4 independent repeats. In (**d-e**), transient transfected DU-L cells with empty or CXCR3-B plasmids. **d** Representative immunoblot of E-cadherin, CXCR3-B and CXCR3, GAPDH as loading control. **e** Semi-quantification of protein expression levels by western blot bands, student t-test, *, *p* < 0.05, *n* = 4. **f**) Quantitative real-time PCR analysis, data shown as ratio of mRNA levels of CXCR3-B/empty plasmids transfections. One representative experiment of 4 independent repeats is presented

Next, to determine whether CXCR3 isoforms may play a role in mediating cancer cell phenotypic changes, CXCR3-A and CXCR3-B were exogenously expressed in DU-H and DU-L cells, respectively. DU-H cells were transfected with pTarget plasmid containing CXCR3-A cDNA. There is no CXCR3-A specific antibody available due to almost complete overlap of the amino acid sequence with CXCR3-B. Thus, we opted to verify CXCR3-A overexpression at the protein level using total CXCR3 antibody. Forced expression of CXCR3-A downregulated E-cadherin protein levels (Fig. 4a and b). Transfection increased CXCR3A mRNA level without statistically affecting CXCR3-B and E-cadherin mRNA levels (Fig. 4c), indicating post-transcriptional regulation.

In the second part of the experiment, CXCR3-B was overexpressed using pTarget-CXCR3-B plasmid in DU-L cells. CXCR3-B overexpression increased E-cadherin protein level significantly (Fig. 4d and e). Again, the cells containing CXCR3-B expressing plasmids showed dramatic elevation of CXCR3-B mRNA but not CXCR3-A mRNA (Fig. 4f). Similar to CXCR3-A, CXCR3-B did not influence E-cadherin mRNA transcription (Fig. 4f). As CXCR3-B is suppressive in cancer cells [25] [29], only a limited overexpression could be achieved in cultured cells. Due to lack of CXCR3-B antibody for immunofluorescence to validate the expression efficiency, additional plasmid pCMV-CXCR3B-DDK was applied to introduce CXCR3B-DDK fusion protein into the DU-L cells. Anti-DDK immunofluorescence shows a limited but reproducible increase in protein expression levels of ddk-tagged-CXCR3-B in DU-L (Additional file 1: Figure S2a), which is consistent with its cell cycle arrest function. Whole protein analysis showed elevated CXCR3B level (Additional file 1: Figure S2b and c), replicating our results with pTarget-CXCR3-B plasmid (Fig. 4d and e). Similarly, no statistically changes of CXCR3A and E-cadherin mRNA levels were found in CXCR3-B overexpressed cells (Additional file 1: Figure S2d). These results indicated the differential regulation roles of CXCR3 variants on E-cadherin expression. Further, the negligible effect on E-cadherin mRNA levels suggests that CXCR3 signaling alters E-cadherin presentation at the post-transcriptional level.

The activation of the CXCR3 isoforms was studied by triggering the receptors with CXCL10/IP-10 or inhibiting with AMG-487. E-cadherin expression was determined in DU-H cells, with predominantly expressing CXCR3-B isoform (Additional file 1: Figure S1b) [25]. Exposure to CXCR3 ligand IP-10, slightly increased, whereas CXCR3 inhibitor AMG-487 mildly decreased E-cadherin in DU-H (Additional file 1: Figure S3a and b). Given CXCR3-A and CXCR3-B differentially regulate E-cadherin in tumors, those minor shift of E-cadherin expression is expected. Interestingly, exposure to the combination of IP-10 and AMG-487 decreased E-cadherin further, due to the additional reduction of membrane-binding CXCR3-B (Additional file 1: Figure S3a, b and c).

### CXCR3-B presentation on PCa tumor cells switched with tumor progression

**Fig. 5:**
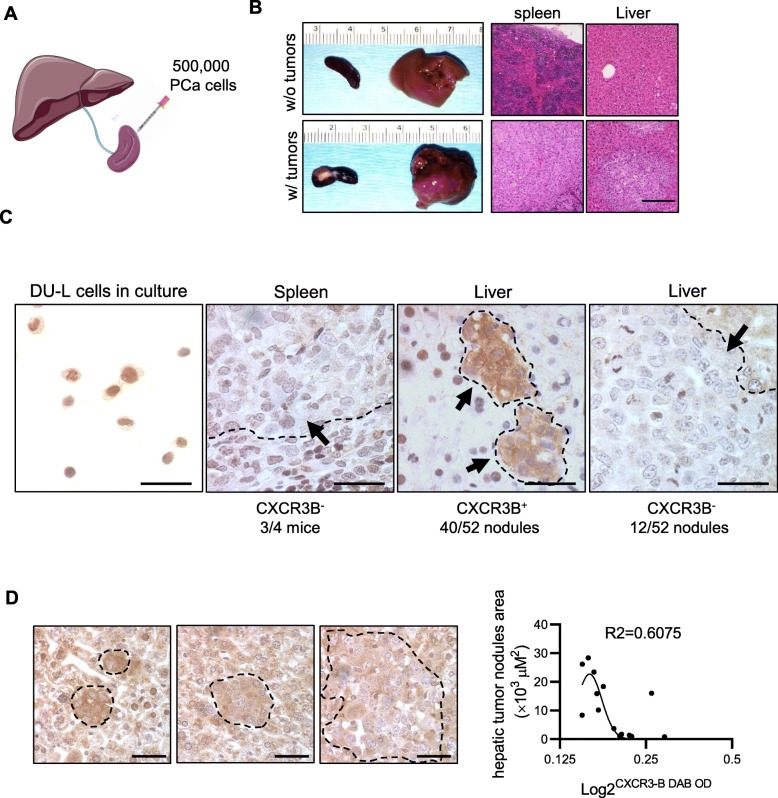
Dynamic expression of CXCR3-B during tumor cells dissemination. **a** Schematic of intrasplenic injection model with spontaneous metastasis to the liver. **b** Representative spleen and liver images and H&E staining with or without tumors after PCa cells inoculation (mice *N* = 6). Bar = 250 μm. **c** Representative IHC staining of CXCR3-B in splenic and hepatic tumors. Tumor area is outlined and pointed by black arrow. Bar = 50 μm. **d** Representative of IHC staining of CXCR3-B in different size of hepatic tumor nodules, all from the same liver section, tumor area is outlined. The correlation of tumor nodules area and semi-quantitative CXCR3-B expression levels is on the right. Bar = 50 μm. Nonlinear fit line with R-squared value has been shown

To further investigate the role of CXCR3-B during tumor metastasis, we inoculated human PCa cells into the spleen of NOD-SCID mice to allow for spontaneous metastasis to the liver (Fig. 5a) [11]. DU145 (DU-L) cells grew in the spleen and disseminated to the liver (Fig. 5b). The expression of CXCR3-B was assessed with immunohistological staining with an isoform-specific antibody. The primary splenic tumor was devoid of CXCR3-B in 3 out of 4 mice, as the cells were low level positive in culture (Fig. 5c), consistent with the above results (Fig. 2d and f). CXCR3-B was presented on the liver metastatic tumor in 40 out of 52 nodules, with the other 12 tumors having low or negligible staining of CXCR3-B. The correlation of CXCR3-B expression level and tumor nodules area was analyzed semi-quantitatively by measuring the CXCR3-B staining. The intensity of staining for CXCR3-B was downregulated as tumor nodule size increased, suggesting a negative correlation between CXCR3-B and the size of metastatic nodules (Fig. 5d).

### CXCR3-B expression in liver metastases correlated with E-cadherin

**Fig. 6:**
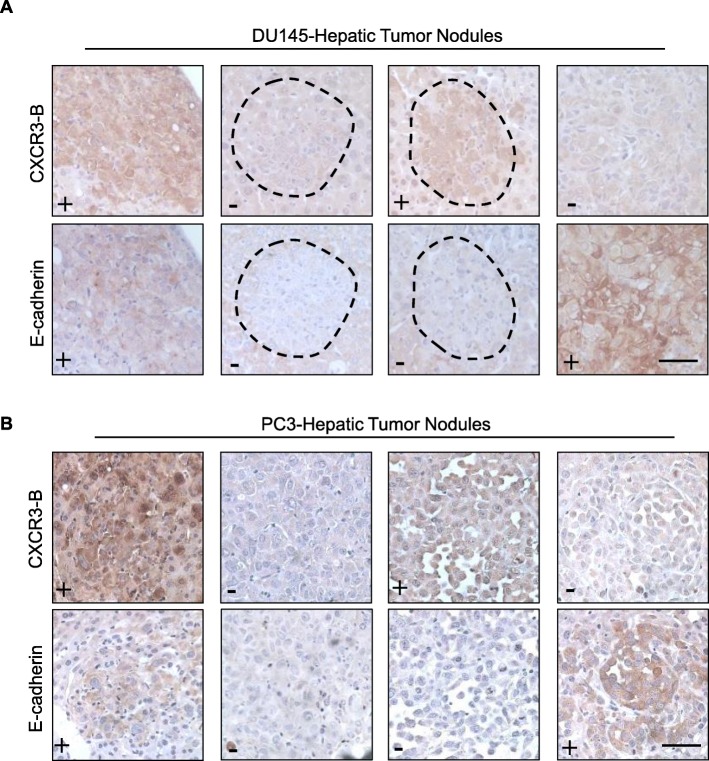
CXCR3-B expression correlates with E-cadherin in PCa liver metastases. DU145 or PC3 PCa cell lines were inoculated via intrasplenic injection in NOD-SCID mice. **a** Representative IHC staining of E-cadherin and CXCR3-B in DU145 cell liver metastases of all four presentations, prevalence of presentation is noted below images and in Tables. **b** Representative IHC staining of E-cadherin and CXCR3-B in PC3 cell liver metastases. +, positive; −, negative. Bar = 50μm. Six mice were injected with DU145 and four mice with PC3

Aggressive prostate cells and tissues, which typically lack E-cadherin expression, have been previously reported to express low level of CXCR3-B relative to CXCR3-A [25]. We reported that hepatocytes can induce E-cadherin expression, at least transiently in the micro-metastatic nodules, such induction also inversely related to the size of the tumor nodule [7, 9, 11]. Thus, based on those and the in vitro data we observed above, we hypothesized that a concomitant expression pattern existed between E-cadherin and CXCR3-B. To test this hypothesis, sister sections of mice liver tissue harboring metastatic PCa tumors were stained with E-cadherin and CXCR3-B respectively. E-cadherin-positive nodules were predominantly CXCR3-B expressing (> 90%), though all the possible combinations of E-cadherin±/CXCR3-B ± (negative means no or low signals) were noted (Fig. 6a, Table 1). A similar but slightly weaker association was also observed for PC3 liver metastases (Fig. 6b, Table 2), as the emergent expression of CXCR3-B is less penetrant in PC3 cells.

**Table 1:**
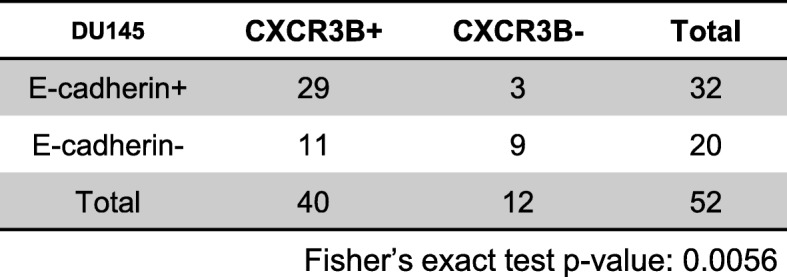
Correlation of CXCR3-B and E-cadherin in DU145 induced hepatic tumor nodules

Fisher’s exact test *p*-value: 0.0056

**Table 2:**
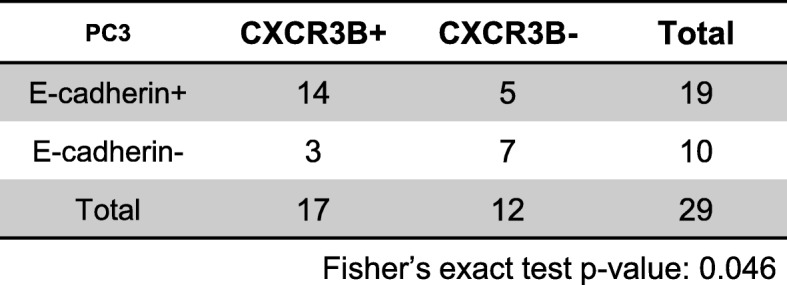
Correlation of CXCR3-B and E-cadherin in PC3 induced hepatic tumor nodules

Fisher’s exact test *p*-value: 0.046

### Coordinate expression of E-cadherin and CXCR3-B in human prostate tumor and metastases

**Fig. 7:**
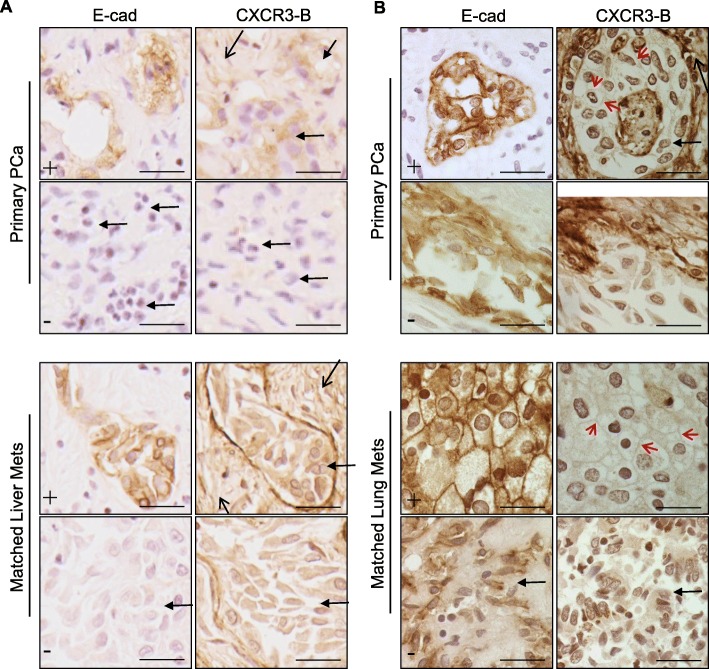
E-cadherin and CXCR3-B IHC staining of patients’ primary prostate tumors and paired metastases. **a** Representative images of E-cadherin (E-cad) and CXCR3-B staining from paired primary and liver metastatic PCa. E-cadherin and CXCR3-B staining were performed on sister sections, the same areas were imaged. Representative E-cad positive (+) or negative (or low, −) images from same section are shown. **b** Representative images of E-cadherin (E-cad) and CXCR3-B staining from paired primary and lung metastatic PCa. Representative E-cad positive (+) or negative (or low, −) images from the same section are shown. Black arrows, tumor stroma; Black arrows with solid triangle head, tumor area; Red arrows point out the membrane staining of CXCR3-B. Bar = 25μm

Paired primary and metastatic prostate tumors autopsy samples from patients were stained with E-cadherin and CXCR3-B in sister sections. E-cadherin was found to be expressed on the membrane in early stage primary tumors, concomitant with CXCR3-B (Fig. 7a and b, top panel). However, E-cadherin was at lower levels in highly differentiated prostate tumors, along with the absence of CXCR3-B on the membrane and reduced cytoplasmic levels (Fig. 7a and b, second panel). In the liver metastases, PCa tumors showed E-cadherin dependent on metastatic stage, with the small dormant micrometastases presenting E-cadherin and the larger aggressive outgrowth devoid of E-cadherin; CXCR3-B was present generally along with the detection of E-cadherin (Fig. 7a). To be notable, CXCR3-B was highly expressed in the stromal cells in both primary prostate cancer (Fig. 7a and b top two panels) and hepatic metastases (Fig. 7 a, bottom two panels). In lung metastases, both E-cadherin and CXCR3-B presented membrane staining in differentiated tumor nodules, and were downregulated in undifferentiated tumors (Fig. 7b). Moreover, the cellular location of CXCR3-B was consistent with E-cadherin in both primary and metastatic PCa. Taken together, E-cadherin and CXCR3-B were positively correlated on both expression levels and localization.

## Discussion

CXCR3 signaling has been implicated in tumor progression with a model emerging that signaling through the CXCR3-A isoform imparts migration and thus dissemination for the mesenchymal cells, whereas in the non-aggressive epithelial cells the CXCR-B isoform would block motility [16]. While this can account for the cEMT and initial escape, this framework does not address the phenotypic shifts during the totality of tumor progression [3]. The model could be valid if the CXCR3 isoform also switches as the phenotype changes from the cEMT through the cMErT to the final emergence cEMT. Herein, we found the CXCR3 isoform dominance shifts during cancer associated phenotypic shifts. In prostate cancers, CXCR3-B predominates in the precursor epithelial cells and in non-invasive early neoplasia, but shifts to CXCR3-A during cEMT; this coincides with a change of ligand effects on cell motility from suppressing to augmenting locomotion. Forced overexpression of the CXCR3-B isoform in the aggressive DU-145 or PC3 cells blocked ligand-induced cell migration [25]. This suggested a role for CXCR3 signaling in reinforcing the behavior of a particular tumor cell phenotype.

This shift of CXCR3 isoform usage appeared to loosely parallel that of E-cadherin, the main and only consistent marker of the epithelial phenotype in carcinoma cells during progression, which is down-regulated to variable degrees during both primary and secondary EMT of cancer progression. Similarly, E-cadherin is down-regulated in high grade tumors and metastatic cancer cells [30] and re-expression of E-cadherin also reduced or blocked cancer migration [31]. Thus, we asked if these two are functionally interlinked.

Herein, we, for the first time, demonstrated that CXCR3-B expression both regulated and was regulated by E-cadherin expression. We showed that CXCR3-A overexpression reduced E-cadherin while CXCR3-B overexpression led to E-cadherin up-regulation. Either E-cadherin or CXCR3-B have been considered as tumor suppressors, acting as cell cycle arresters and thus inhibiting tumor cells proliferation [32–34]. However, during the early stage of tumor metastases, single or small clusters of tumor cells undergo a quiescent dormancy to survive in or adapt to the inhospitable ectopic microenvironments [35, 36]. E-cadherin has been identified a key regulator of this stage wherein it is re-expressed in tumor cells, enables cell dormancy, and provides survival signals to cell death [4, 5, 11]. Similarly, as it is also expressed in these small micrometastases, CXCR3-B might be involved in the early stage of metastases adaptations. However, it is unclear the underlying mechanism. The presented study suggests that at least CXCR3-B plays its role through modulating E-cadherin expression and localization. It is unknown yet whether CXCR3-B and E-cadherin synergetic promotes micro-metastases dormant or survival, or as dependent or independent signaling pathway.

Previous works on CXCR3-B isoform mainly focused on its roles in limiting cancer growth and proliferation or in inducing cancer cell death [32, 33]. This is the first study to establish the correlation between CXCR3-B and E-cadherin for tumor metastasis. However, there are several results that warrant further discussion and validations. First of all, we did not observe elevated E-cadherin gene expression following CXCR3-B overexpression. In cancer cells, E-cadherin expression is regulated via at least three known mechanisms, DNA methylation, transcriptional repression, and signal-induced destabilization and internalization [5]. In prostate cancer cells, E-cadherin is mainly post-transcriptionally regulated wherein autocrine EGFR activation leads to catenin phosphorylation and junctional complexes instability and subsequent E-cadherin internalization and degradation [8, 28]. We are currently investigating the molecular mechanism for CXCR3-B-mediated E-cadherin re-expression. Based on the post-transcriptional effects, we postulate that CXCR3-B inhibits catenin phosphorylation to maintain the stability of junctional complexes. Secondly, the translation of these findings to other cancer types may be limited to certain cancer cell type as E-cadherin promoter hypermethylation could be the main mechanism for E-cadherin downregulation in other metastatic cancer cells such as breast cancer [37]. Our preliminary finding showed that CXCR3-B overexpression did not affect E-cadherin level in MDA-MB-231 breast cancer cells, in which the E-cadherin promoter is hypermethylated, at either mRNA and protein levels (Additional file 1: Figure S4a and b). Current data, however, are inconclusive as direct measurement of promoter methylation in the CXCR3-B overexpressing cells is still pending and the timescale for loss of promoter methylation may be quite extensive [9].

## Conclusions

We report a novel association between CXCR3 isoform and E-cadherin expression, wherein the expression of E-cadherin and CXCR3-B are mechanistically linked to each other, but diametrically affected by CXCR3-A expression. During liver colonization and the initial cMErT, both CXCR3-B and E-cadherin are up-regulated. This correlation may improve chemoresistance and is postulated to also enhance cancer seeding. Further investigations are required to truly establish the mechanism(s) and the functional effects of CXCR3-B overexpression in cancer cells. Since cancer escape from primary sites occurs early during tumorigenesis, specific inhibition of CXCR3-B may be useful to block liver or distant organ colonization.

## Supplementary information

**Additional file 1: Figure S1.** A) Western blot of total-CXCR3 in DU145 treated with 500 nM PD153035 for conversion (PD(MErT)), DMSO was added as control (Ve). B) Western blot of total-CXCR3 in DU-L, DU-H, DU-H E-cadherin knockdown (DU-H-shEcad) cells. C) Average basal cAMP levels in DU-L and DU-H determined using ELISA. Isobutylmethylxanthine (IBMX) was incubated with all groups, and Forskolin (Fsk) treatment served as the positive control (*n* = 3). **Figure S2.** A) Immunofluorescence of DU-L transfected with pCMV-CXCR3B-ddk plasmid (pCMV BOX) and stained with Flag antibody. Bar = 50 μm. B) Western blot of E-cadherin and CXCR3-B in overexpressed cells. C) Protein fold change of CXCR-B and E-cadherin in western blot. D) Fold-change of CXCR3A, CXCR3B and E-cadherin mRNA in DU-L-BOX (data shown as mean + SEM, *n* = 4). **Figure S3.** IP-10 and AMG-487 differentially regulate E-cadherin in DU-H cells. A) Western blot of E-cadherin in DU-H cells treated with 10 ng/ml IP-10, 50 nM AMG-487 or both for 24 h. GAPDH as loading control. B) Immunofluorescence of E-cadherin (green) and DAPI. C) Representative flow assay of membrane CXCR3-B in DU-H cells. The percentage of CXCR3-B^Neg/medium/high^ in gated cells is shown in the right panel. D) Representative flow assay of membrane CXCR3 in DU-H cells. The percentage of CXCR3^Neg/medium/high^ in gated cells is shown in the right panel. **Figure S4.** Overexpression of CXCR3B in MDA-MB-231 breast cancer cells does not promote E-cadherin expression. A) Representative immunoblots of MB-lipo and MB-BOX pTarget (*n* = 3). B) mRNA fold-change for CXCR3A, CXCR3B and E-cadherin normalized to GAPDH. Data shown as mean + SEM, *n* = 3. **Figure S5.** E-cadherin and CXCR3-B expression in primary and metastatic PCa patients. A) Representative images of E-cadherin (E-cad) and CXCR3-B staining from paired primary and bone metastatic PCa. B) Representative images of E-cadherin (E-cad) and CXCR3-B staining from paired primary and bone marrow metastatic PCa.

## Data Availability

Not applicable.

## Abbreviations

DU-H: DU145 sub-line with high E-cadherin
DU-L: DU145 sub-line with low E-cadherin
EMT: Epithelial to mesenchymal transition
MErT: Mesenchymal to epithelial reverting transition
PCa: Prostate Cancer
