# Supplementary material for: Expression of E-cadherin and specific CXCR3 isoforms impact each other in prostate cancer
Source: Cell Commun Signal. 2019 Dec 12;17:164. doi: 10.1186/s12964-019-0489-1 (PMC6909607; doi:10.1186/s12964-019-0489-1)
Supplement: Supplementary file 1 — Additional file 1: Figure S1. A) Western blot of total-CXCR3 in DU145 treated with 500 nM PD153035 for conversion (PD(MErT)), DMSO was added as control (Ve). B) Western blot of total-CXCR3 in DU-L, DU-H, DU-H E-cadherin knockdown (DU-H-shEcad) cells. C) Average basal cAMP levels in DU-L and DU-H determined using ELISA. Isobutylmethylxanthine (IBMX) was incubated with all groups, and Forskolin (Fsk) treatment served as the positive control (n = 3). Figure S2. A) Immunofluorescence of DU-L transfected with pCMV-CXCR3B-ddk plasmid (pCMV BOX) and stained with Flag antibody. Bar = 50 μm. B) Western blot of E-cadherin and CXCR3-B in overexpressed cells. C) Protein fold change of CXCR-B and E-cadherin in western blot. D) Fold-change of CXCR3A, CXCR3B and E-cadherin mRNA in DU-L-BOX (data shown as mean + SEM, n = 4). Figure S3. IP-10 and AMG-487 differentially regulate E-cadherin in DU-H cells. A) Western blot of E-cadherin in DU-H cells treated with 10 ng/ml IP-10, 50 nM AMG-487 or both for 24 h. GAPDH as loading control. B) Immunofluorescence of E-cadherin (green) and DAPI. C) Representative flow assay of membrane CXCR3-B in DU-H cells. The percentage of CXCR3-BNeg/medium/high in gated cells is shown in the right panel. D) Representative flow assay of membrane CXCR3 in DU-H cells. The percentage of CXCR3Neg/medium/high in gated cells is shown in the right panel. Figure S4. Overexpression of CXCR3B in MDA-MB-231 breast cancer cells does not promote E-cadherin expression. A) Representative immunoblots of MB-lipo and MB-BOX pTarget (n = 3). B) mRNA fold-change for CXCR3A, CXCR3B and E-cadherin normalized to GAPDH. Data shown as mean + SEM, n = 3. Figure S5. E-cadherin and CXCR3-B expression in primary and metastatic PCa patients. A) Representative images of E-cadherin (E-cad) and CXCR3-B staining from paired primary and bone metastatic PCa. B) Representative images of E-cadherin (E-cad) and CXCR3-B staining from paired primary and bone marrow metastatic PCa. [file 12964_2019_489_MOESM1_ESM.pptx]

## Slide 1
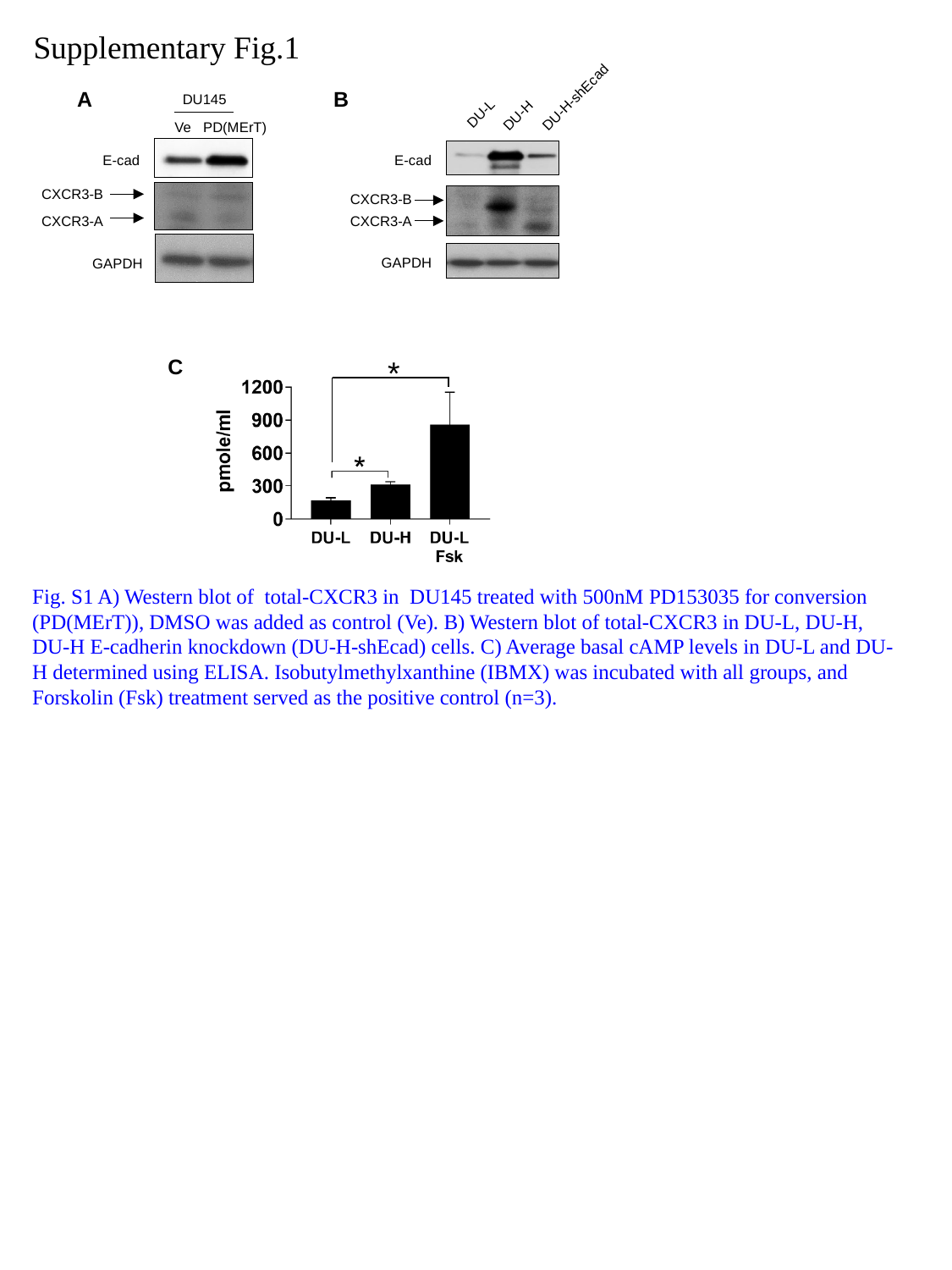

DU-H-shEcad
DU-L
DU-H
E-cad
CXCR3-B
CXCR3-A
GAPDH
Supplementary Fig.1
A
B
DU145
Ve PD(MErT)
E-cad
CXCR3-B
CXCR3-A
GAPDH
C
*
Fig. S1 A) Western blot of total-CXCR3 in DU145 treated with 500nM PD153035 for conversion (PD(MErT)), DMSO was added as control (Ve). B) Western blot of total-CXCR3 in DU-L, DU-H, DU-H E-cadherin knockdown (DU-H-shEcad) cells. C) Average basal cAMP levels in DU-L and DU-H determined using ELISA. Isobutylmethylxanthine (IBMX) was incubated with all groups, and Forskolin (Fsk) treatment served as the positive control (n=3).

## Slide 2
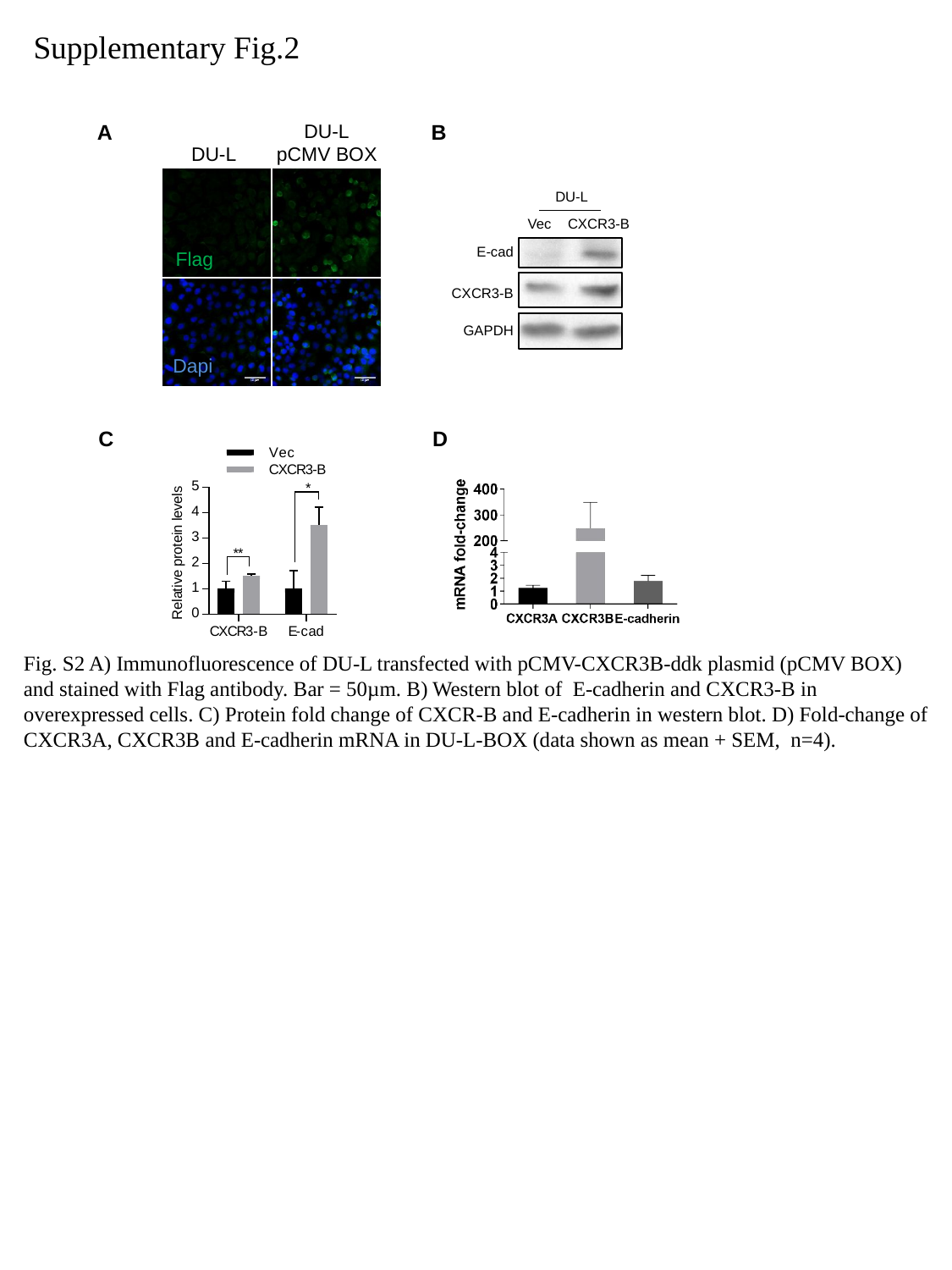

Supplementary Fig.2
A
DU-L
pCMV BOX
DU-L
Flag
Dapi
B
DU-L
Vec
CXCR3-B
E-cad
CXCR3-B
GAPDH
C
D
Fig. S2 A) Immunofluorescence of DU-L transfected with pCMV-CXCR3B-ddk plasmid (pCMV BOX) and stained with Flag antibody. Bar = 50µm. B) Western blot of E-cadherin and CXCR3-B in overexpressed cells. C) Protein fold change of CXCR-B and E-cadherin in western blot. D) Fold-change of CXCR3A, CXCR3B and E-cadherin mRNA in DU-L-BOX (data shown as mean + SEM, n=4).

## Slide 3
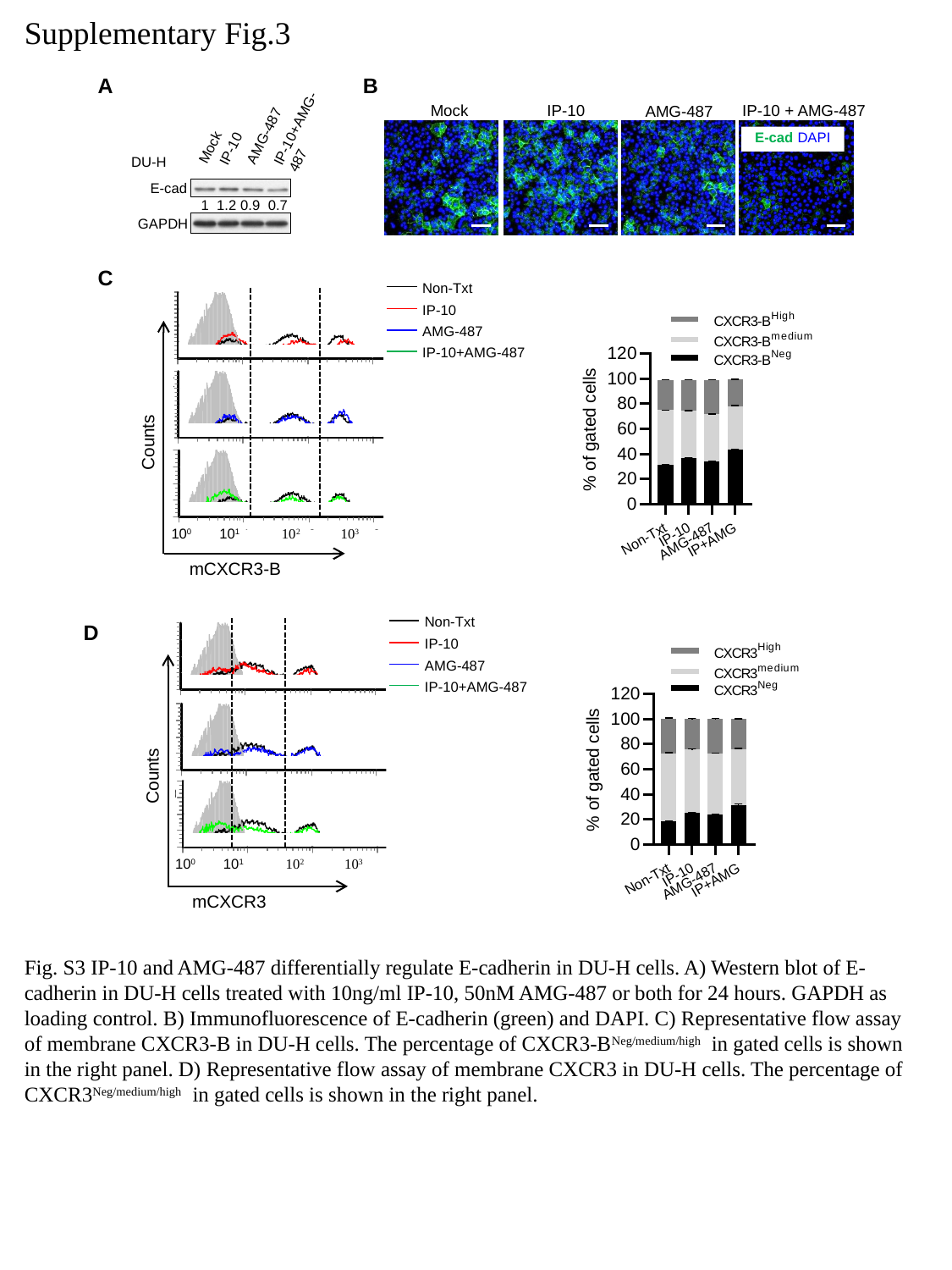

Supplementary Fig.3
A
B
IP-10+AMG-487
AMG-487
IP-10
Mock
DU-H
E-cad
1 1.2 0.9 0.7
GAPDH
Mock
IP-10
IP-10 + AMG-487
AMG-487
E-cad DAPI
C
Non-Txt
IP-10
AMG-487
IP-10+AMG-487
Counts
mCXCR3-B
100 101 102 103
Non-Txt
IP-10
AMG-487
IP-10+AMG-487
D
Counts
mCXCR3
100 101 102 103
Fig. S3 IP-10 and AMG-487 differentially regulate E-cadherin in DU-H cells. A) Western blot of E-cadherin in DU-H cells treated with 10ng/ml IP-10, 50nM AMG-487 or both for 24 hours. GAPDH as loading control. B) Immunofluorescence of E-cadherin (green) and DAPI. C) Representative flow assay of membrane CXCR3-B in DU-H cells. The percentage of CXCR3-BNeg/medium/high in gated cells is shown in the right panel. D) Representative flow assay of membrane CXCR3 in DU-H cells. The percentage of CXCR3Neg/medium/high in gated cells is shown in the right panel.

## Slide 4
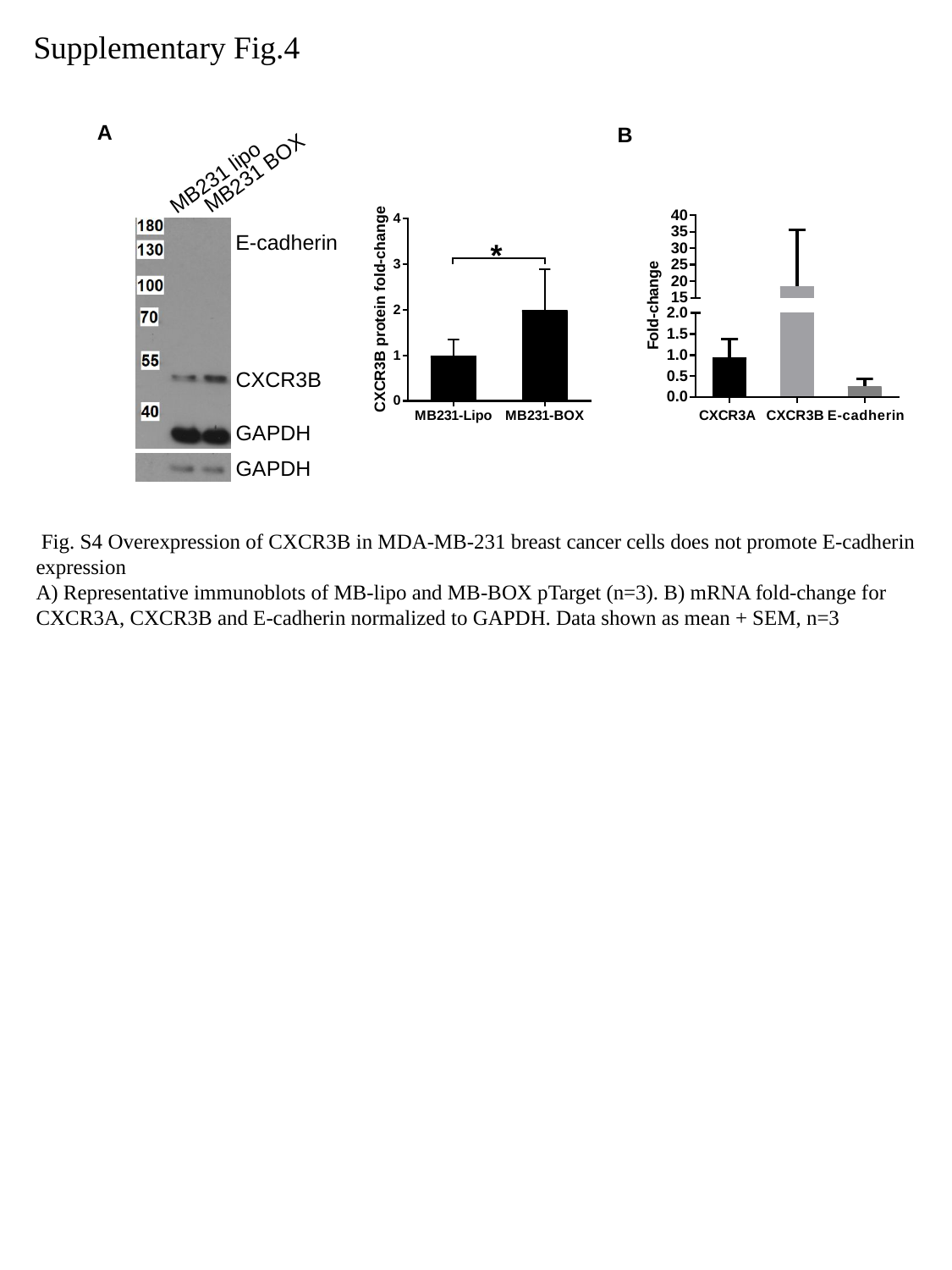

Supplementary Fig.4
A
B
MB231 BOX
MB231 lipo
E-cadherin
CXCR3B
GAPDH
GAPDH
 Fig. S4 Overexpression of CXCR3B in MDA-MB-231 breast cancer cells does not promote E-cadherin expression
A) Representative immunoblots of MB-lipo and MB-BOX pTarget (n=3). B) mRNA fold-change for CXCR3A, CXCR3B and E-cadherin normalized to GAPDH. Data shown as mean + SEM, n=3

## Slide 5
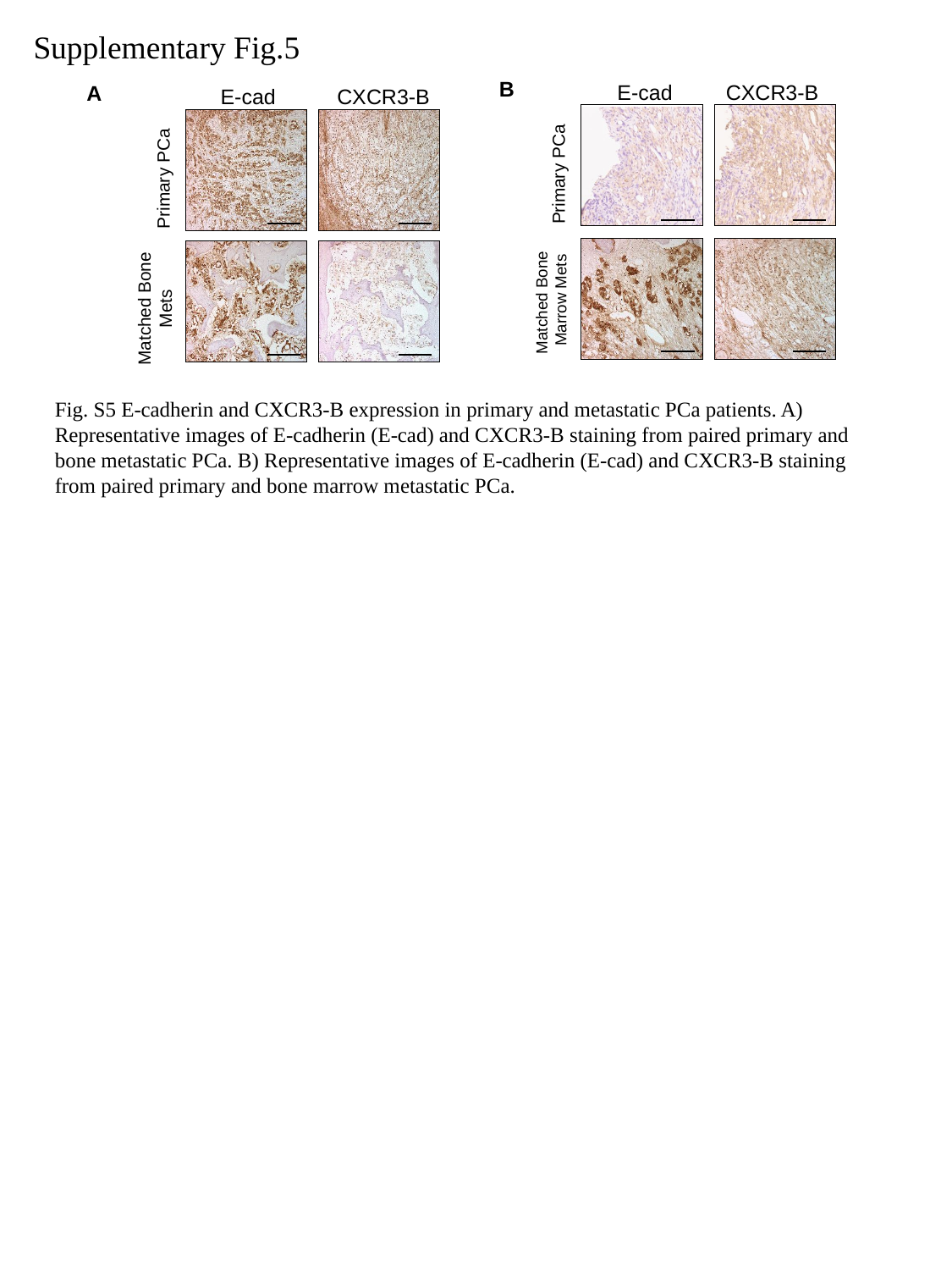

Supplementary Fig.5
Primary PCa
Matched Bone
 Marrow Mets
E-cad
CXCR3-B
E-cad
CXCR3-B
Primary PCa
Matched Bone Mets
B
A
Fig. S5 E-cadherin and CXCR3-B expression in primary and metastatic PCa patients. A) Representative images of E-cadherin (E-cad) and CXCR3-B staining from paired primary and bone metastatic PCa. B) Representative images of E-cadherin (E-cad) and CXCR3-B staining from paired primary and bone marrow metastatic PCa.
